# *Citrus limon var. pompia Camarda var. nova*: A Comprehensive Review of Its Botanical Characteristics, Traditional Uses, Phytochemical Profile, and Potential Health Benefits

**DOI:** 10.3390/nu16162619

**Published:** 2024-08-08

**Authors:** Anna Maria Posadino, Paola Maccioccu, Ali H. Eid, Roberta Giordo, Gianfranco Pintus, Grazia Fenu

**Affiliations:** 1 Department of Biomedical Sciences, University of Sassari, Viale San Pietro 43B, 07100 Sassari, Italy; amposadino@uniss.it (A.M.P.); p.maccioccu@phd.uniss.it (P.M.); gfenu@uniss.it (G.F.); 2 Department of Basic Medical Sciences, College of Medicine, QU Health, Qatar University, Doha 2713, Qatar; ali.eid@qu.edu.qa; 3 Department of Medical Laboratory Sciences, College of Health Sciences, Sharjah Institute for Medical Research, University of Sharjah, Sharjah 27272, United Arab Emirates

**Keywords:** *Pompia*, botanical, traditional uses, phytochemical analysis

## Abstract

*Citrus limon var. pompia Camarda var. nova*, commonly known as pompia, is a distinctive citrus ecotype native to Sardinia, notable for its unique botanical, phytochemical, and potential health benefits. It holds cultural significance as a traditional food product of Sardinia, recognized by the Italian Ministry of Agricultural Food and Forestry Policies. This comprehensive review examines pompia’s traditional uses, taxonomic classification, pomological characteristics, phytochemical profile, and potential health benefits. Pompia phytochemical analyses reveal a rich composition of flavonoids and terpenoids, with notable concentrations of limonene, myrcene, and various oxygenated monoterpenes. Pompia essential oils are primarily extracted from its peel and leaves. Peel essential oils exhibit a high concentration of the monoterpene limonene (82%) and significantly lower quantities of myrcene (1.8%), geranial (1.7%), geraniol (1.5%), and neral (1.4%). In its rind extract, flavanones such as naringin (23.77 µg/mg), neoeriocitrin (46.53 µg/mg), and neohesperidin (44.57 µg/mg) have been found, along with gallic acid (128.3 µg/mg) and quinic acid (219.67 µg/mg). The main compounds detected in the essential oils from pompia leaves are oxygenated monoterpenes (53.5%), with limonene (28.64%), α-terpineol (41.18%), geranial (24.44%), (E)-β-ocimene (10.5%), linalool (0.56%), and neryl acetate (13.56%) being particularly prominent. In pompia juice, the presence of phenolic compounds has been discovered, with a composition more similar to lemon juice than orange juice. The primary flavonoid identified in pompia juice is chrysoeriol-6,8-di-C-glucoside (stellarin-2) (109.2 mg/L), which has not been found in other citrus juices. The compound rhoifolin-4-glucoside (17.5 mg/L) is unique to pompia juice, whereas its aglycone, rhoifolin, is found in lemon juice. Other flavonoids identified in pompia juice include diosmetin 6,8-C-diglucoside (54.5 mg/L) and isorhamnetin 3-O-rutinoside (79.4 mg/L). These findings support the potential of pompia in developing nutraceuticals and natural health products, further confirmed by its compounds’ antioxidant, anti-inflammatory and antibacterial properties. Future research should focus on optimizing extraction methods, conducting clinical trials to evaluate efficacy and safety, and exploring sustainable cultivation practices. The potential applications of pompia extracts in food preservation, functional foods, and cosmetic formulations also warrant further investigation. Addressing these areas could significantly enhance pompia’s contribution to natural medicine, food science, and biotechnology.

## 1. Introduction

*Citrus limon var. pompia Camarda var. nova*, commonly known as pompia, is a distinctive citrus ecotype native to the Barony, a historical region in Central–Eastern Sardinia, Italy. The earliest information about pompia was documented by Moris in the 19th century [1]. Pompia is notable for its unique botanical characteristics, rich phytochemical composition, and potential health benefits.

Currently, The Plant List (http://www.theplantlist.org) (accessed on 1 July 2024) and The International Plant Names Index (http://www.ipni.org) (accessed on 1 July 2024) do not assign a specific botanical name to pompia, following extensive debate on its correct classification. Although citrus fruits were described in Sardinia as early as 500 BCE, pompia was first defined in 1837 as *Citrus medica monstruosa* due to the unusual shape of its fruit [1]. However, the unique morphological, phytochemical, and genetic characteristics of pompia, along with its close affinities to the *Citrus limon* s.l. complex—particularly its polyembryony and generally fertile seeds—led Camarda et al. [2] to reject the initial classification of this citrus as a cultivar of citron (*Citrus medica* L.). Thus, these authors attributed a new taxonomical classification to pompia, naming it *Citrus limon var. pump Camarda var. nova*, thereby recognizing it as a lemon cultivar rather than a variety of citron. In this context, Mignani et al. [3] highlighted the similarity of pompia fruit to *Citrus limon* (L.) *Osbeck* and *Citrus medica* L. This evaluation, based on genetic analysis, revealed a remarkable similarity between pompia and the lemon genotype “Zagara Bianca” as well as the citron “Etrog”. On the other hand, Petretto et al. [4], studying the volatile molecules in various citrus samples, classified pompia as most closely related to *Citrus sinensis*, *Citrus paradisi*, and *Citrus aurantium*. In contrast, citron and lemon were the most distant regarding the emitted volatile organic compounds (VOCs). In the study of Curk et Luro (2018) [5], a genetic marker evaluation of citrus fruits classified pompia as a hybrid between *Citrus m* and *Citrus aurantium*. Later, Deiana et al. [6] studied the Volatile Organic Compounds (VOCs) emitted from the flavedo extracts of the traditional pompia candied (“Sa *Pompìa* Intrea”). They found that the production methods of this traditional Sardinian candied citrus peel do not influence the composition of volatile molecules derived from the pompia peel. Limonene, the most abundant terpene in the peel, was found to be equally copious (over 70%) in the untreated peels, as also reported by Petretto et al. [4,6].

In 2004, pompia was awarded the Slow Food Presidium (https://www.fondazioneslowfood.com/en/slow-food-presidia/pompia/) (accessed on 1 July 2024) designation as a special ecotype significant to the Sardinian economy due to its popularity among consumers. Therefore, pompia is considered a crucial biodiversity resource that warrants protection.

Pompia is recognized as a Traditional Food Product of Sardinia (P.A.T.) and is included in a list prepared by the Italian Ministry of Agricultural, Food, and Forestry Policies, based on recommendations from the Sardinian Region. This list features products with processing, conservation, and seasoning methods that have been established over at least twenty-five years, following traditional practices.

In this work, we analyze pompia, examining the fruit’s skin, leaf, rind, and juice, as well as describing the plant in regard to its taxonomic and phenotypic aspects to its traditional and modern uses. Additionally, we report on the fruit’s phytochemistry across all its studied parts, along with some potential health benefits.

## 2. Taxonomic Classification

Morphological and genetic analysis confirms the distinct individuality of pompia, which can be attributed to both its varietal status and cultural significance. As a varietal rank, pompia typically reproduces from seed, preserving the essential characteristics of the fruit, including the polyembryony of its seeds, a trait previously noted by D’Aquino et al. [7]. Culturally, pompia is exclusively cultivated and regularly propagated through vegetative methods, primarily grafted onto bitter orange (*Citrus aurantium* L.) or ponciro (*Poncirus trifoliata* L.). Additionally, pompia is confined to a limited geographical region, further emphasizing its distinctiveness [2].

The intra-specific rank is justified by the fact that polyembryony likely originates from somatic cells of the ovum, nucellus, or ovary, which maintain their genetic characteristics [2]. Mignani et al. [3] suggest that the origin and taxonomy of pompia are uncertain, but they note a strong affinity with citron and lemon, considering it a probable hybrid between the two species. According to Camarda et al. [2], pompia shows greater affiliation with the lemon group rather than the citron group, based on its morphological, biological, and genetic characteristics, including polyembryony and pronounced juice acidity.

**Figure 1:**
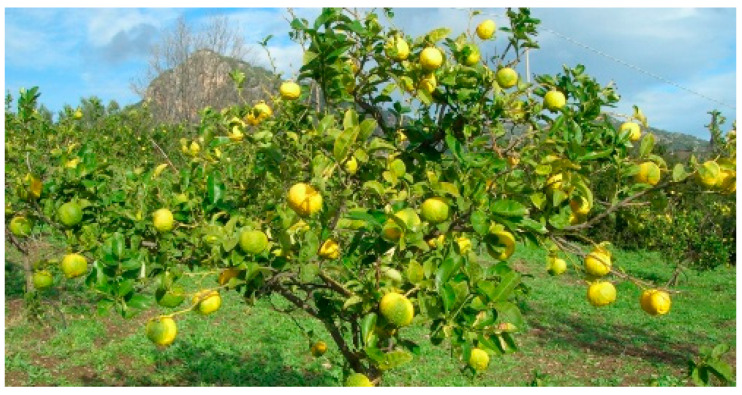
Pompia plants in a citrus grove of the Baronia (https://www.biodiversitasardegna.it/laore/it/agrobiodiversita/repertorio-regionale/risorsa/Pompia/) (accessed on 1 July 2024).

The chemical analysis of pompia essential oil [8,9,10,11,12] highlights the complex phytochemical characteristics, which exhibit relatively consistent differentiation compared to other citrus specie [13]. Based on these factors, it seems reasonable to classify pompia within the group of citrates due to its consistent phytochemical and genetic traits, which are present in agricultural areas limited to regional levels and geographically isolated from one another. These vegetative populations can be considered stable hybrids between lemon and citron, potentially with some contribution from *melangolo*, displaying intermediate characteristics between the two species (Figure 1).

## 3. Pomological Characteristics

The pompia plant is an evergreen sapling, typically 2–3 m tall, with a very branched and expansive habit. D’Aquino et al. [7] evaluated several plant characteristics. The pompia tree (Figure 1) is robust and can grow large if not pruned. The leaves have non-winged petioles (6–10 mm) and a leathery, dark green ovate–lanceolate lamina (5–10 × 3–8 cm) with a pronounced central vein. The shoots are purple, and the flowers are white or light rose. The petals are rounded at the apex, with numerous stamens bearing sessile anthers (15–18 × 4–6 mm). The young branches are greenish and have robust spines (1–4 cm) [2]. The fruit is oblate in shape and, depending on the flowering period and the position of the fruit on the tree, it has a rough and irregular surface and is firmly attached to the tree [2,3].

**Figure 2:**
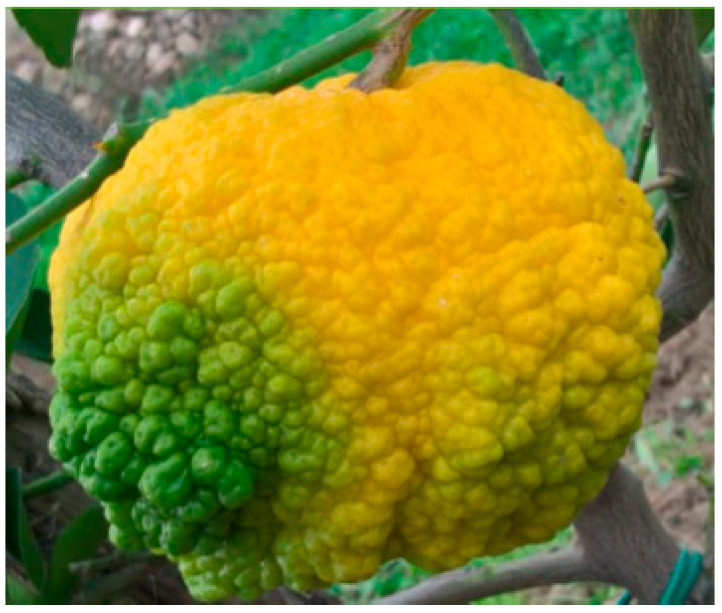
Pompia fruit (https://www.biodiversitasardegna.it/laore/it/agrobiodiversita/repertorio-regionale/risorsa/Pompia) (accessed on 1 July 2024).

The fruit can vary from smooth to wrinkled, with pronounced ribbing and lumps that significantly alter its appearance due to an irregular and abnormal increase in the albedo. The affinity of the var. pompia with the citron is evident in the thickness of the mesocarp and the frequent roughness of the epicarp. However, it differs in the acidity of its juice. Pompia can be classified among the group of lemons known as cedars [3]. Usually, the fruits most exposed to sunlight have an irregular shape, while those further inside the branches are smooth. The average weight of the fruit is around 320 g, but there is considerable variability, with some fruits weighing over 500 g and others less than 200 g. Fruit yield is strongly linked to the fruit size; the yield is low with the largest fruits, which can sometimes reach 700 g each [3]. The rind has an average thickness of 1.5 cm, and the number of segments varies between 11 and 13. There is also significant variability in the number of seeds: some fruits contain 4–5 seeds, while others can have more than 15 seeds, with an average of 10 seeds per fruit [7]. The seeds are polyembryonic, typically containing 3–4 embryos per seed, which classifies pompia among the hybrid fruits, as cedar varieties are monoembryonic (Figure 2) [7].

The peel is hard and can be easily removed only when the fruit is ripe. It is very rich in essential oils, emitting a scent similar to lemon with a fine and fragrant aroma [7].

The shape of the crushed fruit deserves special attention; it is wider than it is long, sometimes with a diameter twice its length. On the same plant, the fruit can vary from quite smooth to wrinkled, with consistent ribbing and lumpiness, which strongly modifies its appearance due to an irregular and abnormal increase in the albedo [2]. The thickness of the mesocarp, one of its main features, is primarily used in the production of candied fruits. In lemon and citron fruits, this characteristic and the typical monstrosity are often caused by an eriophyid mite, *Eriophyes sheldoni Ewing* [2]. However, an analysis conducted by the Pathology and Entomology section of the Agriculture Department at the University of Sassari found no match for this mite in the tested samples. Instead, another mite, *Panonychus citri McGregor*, was sometimes present as a possible cause of fruit malformations [2].

**Figure 3:**
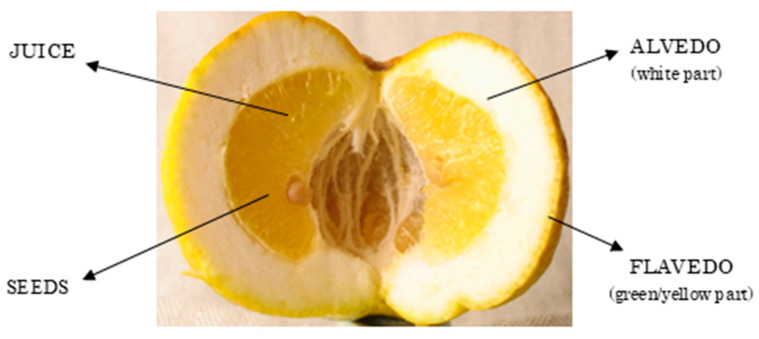
An illustrated photo of opened pompia fruit (https://it.wikipedia.org/wiki/Pompia#/media/File:Pompia_frutto_aperto.jpg) (accessed on 1 July 2024).

To assess the color of pompia peel, a subjective evaluation was performed based on the percentage of the yellow surface. For an objective evaluation, measurements were carried out on 48 pompia fruits using a chromometer at three equatorial points on each fruit, according to the color scale established by the Commission Internationale de l’Eclairage (CIE) (L*, a*, b* color scale, where L* indicates lightness, and a* and b* are chromaticity coordinates) [7]. At the beginning of October, the fruits are still completely green. Objective evaluations of the peel showed a significant increase in the L* value, indicating lightness, followed by minor variations. The a* values, indicating the red–green spectrum, rose progressively over the sampling period, with the final value slightly above 0. The b* component, representing the blue–yellow spectrum, remained positive throughout. By December, objective measurements indicated that the peel color evolution was complete, with final a* and b* values indicating a strong yellow component and a very weak red part. Conversely, the pulp underwent minimal changes, with the a* component remaining negative and the b* component positive, but significantly lower than the peel b* values (Figure 3) [7].

As the fruit matures, its specific weight decreases. The juice content also decreases, dropping from 20.8% in October to 15.5% in March [7]. Additionally, during the sampling period, there is a noticeable decrease in puncture resistance, while deformation increases significantly starting in January. This indicates a lack of correlation between deformation and resistance to perforation [7]. The parameters of fresh pompia fruits, such as pH (2.55), titratable acidity (5.81% citric acid), vitamin C (43.6 mg/100 mL), and total soluble solids (6.22° Brix), are characteristic of lemons [8,9]. During the sampling period, the pH increases from 2.35 to 2.69, while titratable acidity decreases slightly (6.74–5.99% of citric acid), and soluble solids decline from 7.51° to 6.48° Brix. However, the vitamin C content shows the most significant variation during maturation, decreasing from 33.97 to 22.54 mg ascorbic acid/100 mL. These chemical parameters are very close to those of lemons grown in similar environmental conditions [8,9].

Over the ripening period, endogenous CO_2_ levels progressively increase, while endogenous O_2_ levels decrease. Despite significant variability between individual fruits, endogenous C_2_H_4_ shows a general increasing trend, with a final yield of 0.13 µL·L^−1^. As maturation advances, C_2_H_4_ production rises, although the variability between individual fruits remains considerable and statistically inconsistent. The general physiology of pompia is similar to that of lemon fruit. In various experiments with lemon cultivars, similar trends in respiratory activity are observed. The small but gradual decrease in respiration activity intensity, coupled with a rise in endogenous CO_2_ and a reduction in endogenous O_2_, is characteristic of citrus fruits [10,11]. The physiological drop of metabolism due to aging can be interpreted as a result of decreased peel gas permeability, possibly linked to the continuous accumulation of maturation-associated wax on the fruit, which leads to enhanced endogenous CO_2_ levels and reduced respiration activity [14].

## 4. Genotypic Characterization

To establish a foundation for molecular phylogenetic studies, Orrù et al. [12] described the systematic aspects of pompia using biomolecular data. Total RNA was extracted from fresh leaves collected during the driest months (July–September). Genes encoding enzymes primarily expressed in plants near Siniscola, the geographical area where pompia grows, were identified. Additionally, genes abundantly expressed in many plant species living in arid zones were also identified. These genes encode protein products related to antioxidant enzymes, such as superoxide dismutase (SOD, EC 1.15.1.1), catalase (CAT, EC 1.11.1.6), peroxidase (POD, EC 1.11.1.7), and polyphenol oxidase (PPO). The results suggest a clear relationship between the POD sequences of *Citrus sinensis* and *Citrus clementina* and the POD sequence of pompia, which were grouped in the same cluster and distinctly separated from other non-citrus POD sequences.

A study by Luro et al. [13] presented an allelic comparison between citrus species, revealing that pompia is similar to both lemon and Marrakech limonette—a cross between sour orange (*C. aurantium* L.) and citron, with citron likely serving as the pollinator. Two Italian citron varieties, *Diamante* and *Common Poncire*, were identified as potential male parents. However, the study could not differentiate varieties of sour oranges, as varietal diversification in this group results from DNA sequence variations that SSR or InDel markers could not detect. Based on the analysis of fruit, leaf, and seed phenotypes and juice chemical composition, pompia appears to be equally distinct from citron, lemon, and sour orange. At the leaf level, pompia’s essential oil composition is similar to that of citron, while its zest is much closer to that of sour orange.

By conducting flow cytometric and karyological analyses, in their study, Viglietti et al. [15] aimed to understand the implications of certain citrus varieties in relation to pompia fruit. They used Sanger sequencing on chloroplast DNA (cpDNA) barcodes representing the psbA-trnh intergenic region and the trnL intron, along with theInternal Transcribed Spacer (ITS) nuclear locus. The results showed that nucleotide variants, as well as chloroplast and nuclear DNA polymorphisms, indicate that all pompia accessions share a paternal origin with *Citrus medica* and a maternal origin with *Citrus aurantium.*

Based on the complete sequences of nuclear and chloroplast DNA markers, the authors proposed that the cedar “Rhobs el arsa,” used in North Africa (Morocco and neighboring territories) and referred to as “Al-zanbu” and “Koubs el arsa,” represents a single taxonomic and genetic unit. In contrast, analyses using molecular markers such as AFLP (amplified fragment length polymorphisms), RAPD (random amplified polymorphic DNA), and SCAR (sequence characterized amplified region) by Mignani et al. [3] provided evidence of similarity among five citrus species and pompia. The “Zagara Bianca” lemon and the “Etrog” citron were found to be the most similar genotypes to pompia, suggesting a close correlation among them. Additionally, a strong relationship was observed among the different pompia accessions.

## 5. Traditional Uses of Pompia Fruit

Since the dawn of human civilization, traditional medicine has played a crucial role in regulating health and treating illnesses [16,17,18,19,20]. Various parts of citrus, including the leaves, pulp, and peel, have been used for generations in traditional medicine due to their scientifically proven therapeutic potential and safety for human use [21].

**Figure 4:**
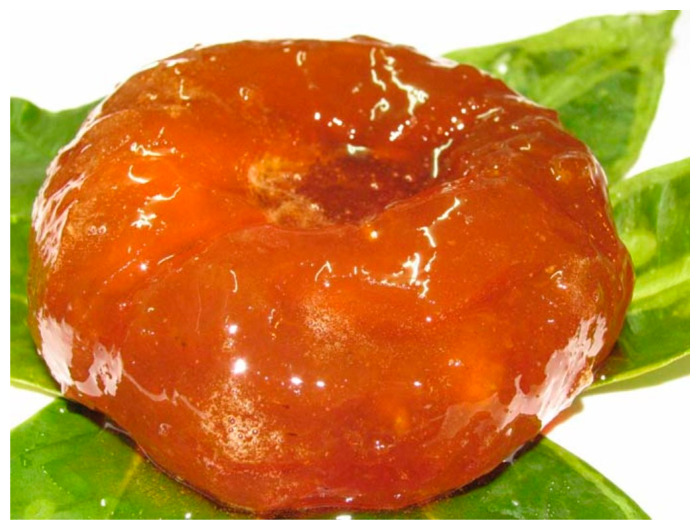
Special Sardinian dessert cake: “Sa Pompia Intrea” (https://it.wikipedia.org/wiki/Pompia#/media/File:Pompia_intrea.jpg) (accessed on 1 July 2024).

The traditional uses of pompia’s fruit have focused on its juice, similar to lemon juice, in culinary traditions. However, pompia juice has a unique taste due to its distinct essential oils. In Sardinian tradition, pompia juice is used as a substitute for lemon juice to add flavor to salads and special dishes. It is particularly appreciated in the form of sliced fruit, which is the main ingredient in traditional Sardinian dessert cakes such as “Sa Pompia Intrea” (where the albedo is slowly cooked in honey) [6], (Figure 4), ”Sa Pompia Prena” (similar to “Pompia Intrea” but with finely chopped almonds added), and “S’Aranzada” (where the pompia rind is caramelized in honey and almonds). The peel is also used in the preparation of fruit cakes after being candied. Additionally, it is used to make slushies, ice creams, and jams. Recently, pompia has been incorporated into craft beers by Sardinian breweries and beer firms. The rind can also be extracted with ethyl alcohol to obtain essential oil, which is used to produce a typical and excellent digestive liqueur known as “Pompinello” [7].

## 6. Phytochemistry of Pompia

### 6.1. Peel Essential Oil

In the study of Fenu et al. [22], pompia was harvested at three different times of the year, February, March, and December. The collected fruits were wiped within one day from harvest. The outer part of the skin was removed by hand with a potato peeler and collected for essential oil extraction. Oil samples were steam distilled for 4 h in a Clevenger-type apparatus. The obtained oils were liquid and transparent, recovered directly from the surface of the distillate without adding any solvent, and stored at −20 °C under a nitrogen atmosphere until GC-MS (Gas Chromatography–Mass Spectrometry) analysis. No surfactant was necessary during storage to keep the oils in solution.

The oil obtained from raw fruits harvested in December contained high amounts of limonene (82.1%) and much lower quantities of myrcene (1.8%), geranial (1.7%), geraniol (1.5%), neral (1.4%), α-pinene (0.7%), and cis-β-ocimene (1.0%) (see Table 1). Essential oil extraction from ripe fruits harvested in February resulted in a slight change in composition. The limonene percentage increased to 82.7%, while most other compounds also showed an increase: myrcene (2.6%), geranial (1.8%), neral (1.6%), and α-pinene (0.9%). However, geraniol (0.5%) and cis-β-ocimene (0.8%) decreased. The oil from aging fruits harvested in March showed a decrease in the limonene percentage (77.5%) in favor of the remaining compounds: myrcene (3.1%), geranial (3.7%), geraniol (1.6%), neral (3.2%), α-pinene (1.0%), and cis-β-ocimene (2.6%).

Limonene, the most concentrated substance in the pompia peel, is a monoterpene that can be extracted from many fruits and vegetables including citruses, tomatoes, and mint. At full fruit maturation, it represented 82.1% of pompia oil compounds, one of the highest concentrations among cedars (see Table 2). However, the pompia peel oil shows uniquely high concentrations of other bioactive substances like α-pinene, myrcene, and cis-β-ocimene, which have not been reported before [23,24]. When considering oils from fruits harvested at different times, the concentration of limonene is highest at complete fruit maturation and decreases in aging fruits. This reduction might reflect both limonene metabolism and water shortage that typically occurs in March in Sardinia, Italy. Water scarcity and high temperatures could lead to limonene evaporation from the fruit skin, resulting in a relative increase in the concentration of other compounds. Cis-β-ocimene and geraniol, which are precursors to the limonene molecule, show a relative decline at complete fruit maturation when limonene concentration is at its peak. Conversely, geranial and neral, which are limonene metabolites, show a constant concentration increase during fruit maturation and aging. Myrcene and α-pinene also increase consistently throughout the maturation and aging process. The roles of geranial and neral in the metabolic pathways of citrus skin remain to be clarified, as they have not been reported as limonene metabolites [24].

In a study by Flamini et al. [25], the authors analyzed the composition of essential oils (EO) obtained by both hydro-distillation (HD) and cold pressing (CP) of pompia peels and leaves collected in November. In the peel EOs, over 80% are monoterpene hydrocarbons. In the CP peel EOs, these hydrocarbons constitute 98.33% of the total EO composition, with limonene being the most represented molecule at 95.77% (Table 1). Limonene is also the most abundant component in the HD peel EO, where its relative quantity exceeds 75% (77.44%). Myrcene is the second most prevalent monoterpene hydrocarbon found in both HD and CP peel EOs. This class of hydrocarbons is also present in the HD leaf EO, with limonene being the most abundant component (Table 1). However, in this sample, the trans-β-ocimene molecule is significantly present, whereas it accounts for less than 1% in both HD and CP peel EOs.

The HD peel EO exhibits a greater variety of molecules compared to the CP peel EO. Notably, oxygenated monoterpenes are present in significant quantities in the HD peel EO (16.44%), while they are only found in trace amounts in the CP peel EO. Geranial and neral are present in considerable amounts in the HD peel EO but only in trace amounts in the CP peel EO. Their derivatives, geranyl and neryl acetates, make up over 4.4% of the HD peel EO, while they are detectable only in very small quantities in the CP peel EO. Conversely, the derived alcohols of geranial and neral are less than 1.5% in the HD peel EO and are absent in the CP peel EO. Sesquiterpene hydrocarbons constitute less than 2.5% in the HD peel EO and 1.62% in the CP peel EO. The CP peel EO is primarily composed of limonene, which accounts for over 95% of the total composition.

**Table 1:** The composition of the rind essential oil from pompia, as studied by Fenu et al. [22] (harvested at three different times of the year), is compared with the work of Flamini et al. [25], who used cold pressing and hydro-distillation for the peels and cold pressing for the leaves. Compounds reaching concentrations of at least 0.45% during the maturation process are indicated. Values are expressed as absolute weight percentages.

Compounds	Fenu et al. Dec.2008 %	Fenu et al. Feb.2009 %	Fenu et al. Mar.2009 %	Flamini et al. Nov.2018 Peel (HD) %	Flamini et al. Nov.2019 Peel (CP) %	Flamini et al. Nov.2019 Leaf (HD) %
Limonene	82.1	82.7	77.5	77.44	95.77	28.64
Myrcene	1.8	2.6	3.1	2.12	1.55	0.91
Geranial	1.7	1.8	3.7		-	
Neral	1.4	1.6	3.2		-	
α-Pinene	0.7	0.9	1.0	0.43	0.51	
Geraniol	1.5	0.8	1.6	1.46	-	
Cis-β-ocimene	1.0	0.8	2.6	-	-	0.46
Trans-β-ocimene				0.99	0.50	10.5
Linalool	-	-	-	1.13	-	0.56
Nerol	-	-	-		-	1.49
Monoterpene hydrocarbons				81.12	98.38	42.01
Oxygenated monoterpenes				16.44	-	53.50
Sesquiterpene hydrocarbons				2.45	1.62	1.44

**Table 2:** Chemical composition of peel essential oils of some citrus samples. Values are expressed as absolute weight percentages.

Compound	*Pompia* [22] %	*Citrus aurantium* [26] %	*Citrus limon* [27] %	*Citrus medica* [28] *%*
Limonene	82.1	77.53	53.9	46.9
Myrcene	1.8	2.76	2.7	1.5
Neral	1.4	-	1.7	2.8
Geranial	1.7	-	2.2	5.4
Geraniol	1.5	-	0.3	0.1
a-Pinene	0.7	2.98	0.7	2
b-O-cimene	1	-	-	0.8

### 6.2. Rind Extract

The extraction of polyphenols from citrus is often performed using a mixture of ethanol and water (1:1 *v*/*v*), as demonstrated by Khan et al. [29], who used an ultrasound-assisted technique to induce the formation of micronized particles and facilitate solute diffusion in the extractive solvent. The ethanol/water mixture was selected for its safety and biocompatibility. Additionally, previous studies have shown that this mixture effectively yields fruit and flower extracts with a high content of phenolics and flavonoids [30,31]. This method is inexpensive, environmentally friendly, and easily reproducible.

Due to the limited literature on the chemical composition of pompia fruit, the major components of the rind extract were determined using HPLC–MS/MS. HPLC techniques are widely employed for the separation and quantification of phenolic compounds in natural extracts [32,33]. When coupled with a diode array detector (DAD), these techniques can simultaneously scan samples at multiple wavelengths, providing the spectral information necessary for compound identification.

For this study, Manconi et al. [34] collected pompia fruit in January 2014, at full maturity, near the Cedrino River in Orosei, eastern Sardinia, Italy. The rind of the fresh fruits was separated using an ethanol/water blend, dried, powdered, and freeze-dried to produce a yellow powder, which was stored under vacuum until use. For quantitative analysis, a sample of the powder was reconstituted in methanol (sample to solvent ratio 1:100 *w*/*v*), filtered, and injected into the analytical system. Due to the complexity of the matrices, the phenolic components of pompia were identified using a combination of HPLC and MS, which is preferred for extract analysis. The following compounds were identified and quantified: eriocitrin, ferulic acid, isoquercetin, isorhamnetin rutinoside, myricitrin, naringin, neoeriocitrin, neohesperidin, phlorizin, quinic acid, robinin, rutin, and sinapic acid. Among these, quinic acid was the major component of the extract, with a concentration of 219.67 µg/mg (Table 3).

Flavanones are well-represented in plants, with high concentrations found in many citrus rind extracts [35]. The primary flavanones in pompia rind are naringin (23.77 µg/mg), neoeriocitrin (46.53 µg/mg), and neohesperidin (44.57 µg/mg). Naringin, the glycoside of naringenin (naringenin-7-neohesperidoside), contributes to the fruit’s bitter taste. Neohesperidin (hesperetin-7-neohesperidoside), along with naringenin and its glycosides, is notable for its presence in foods. Neoeriocitrin, a less common citrus flavanone, is found in high concentrations in *Citrus aurantium* extracts [29,36,37]. The phytochemical profile of pompia rind shows similarities to that of *C. aurantium*, supporting previous findings [4].

In a more recent study [38], the authors harvested pompia fruits at full maturity in January 2015 near the Cedrino River in Orosei, eastern Sardinia. Portions of the powder were dispersed in ethanol and transferred to a semi-automatic extractor. The extractive dispersion was then centrifuged, the ethanol was removed under reduced pressure, and the residue was dispersed in water and freeze dried. The extract was stored under vacuum and protected from light until use. For qualitative and quantitative determinations, the freeze-dried extract was dispersed in methanol and analyzed using HPLC-DAD/MS.

The extract contained gallic acid as the major compound (128.3 µg/mg), followed by neohesperidin (76.5 µg/mg), neoeriocitrin (42.5 µg/mg), eriocitrin (40.4 µg/mg), naringin (28 µg/mg), and hesperidin (16.9 µg/mg) (Table 3).

**Table 3:** Main components of pompia rind extract expressed as µg/mg of dry extract. Values are expressed as µg/mg.

Compound	Manconi et al., 2016 [31] µg/mg	Manconi et al., 2018 [38] µg/mg
Gallic acid	-	128.3
Eriocitrin	0.09	40.4
Neoeriocitrin	46.53	42.5
Naringin	23.77	28
Hesperidin	-	16.9
Neohesperidin	44.57	76.5
Myricetin-3-galactosyde	-	29.3
Ferulic acid	1.03	-
Quinic acid	219.67	-
Robinin	1.08	-
Sinapic acid	30.13	-
Rutin	8.61	-

### 6.3. Leaves Essential Oil

Essential oils are complex mixtures of lipophilic, liquid, and volatile compounds, often terpenoids, found in higher plants. They primarily contain monoterpenes (both hydrocarbon and oxygenated forms), sesquiterpenes (both hydrocarbon and oxygenated forms), as well as phenolic compounds [39].

Fancello et al. [40] analyzed the qualitative and quantitative chemical composition of essential oils from pompia leaves using fruits collected in June near Siniscola, Sardinia, Italy. The essential oils were obtained by steam distillation, yielding a colorless oil with a pleasant aroma. The chemical composition of the essential oils was determined using GC-MS (Gas Chromatography–Mass Spectrometry), while quantitative data were obtained using GC-FID (Gas Chromatography–Flame Ionization Detection). The essential oil components were categorized by their chemical classes, such as monoterpenes and sesquiterpenes, and by their functional groups, including alcohols and esters.

GC-MS analysis revealed the presence of 23 compounds in the essential oil, with limonene being the major component (256 mg/mL), followed by neral (173 mg/mL) (Table 4). Previous studies by Abderrazak, et al. [41] and Ellouze et al. [42] indicated the absence or only trace amounts of limonene in essential oils extracted from citrus aurantium leaves, which is one of the Citrus species showing chemical similarity to pompia (Petretto et al. [4]).

As previously reported, Flamini et al. [25] also analyzed pompia leaf essential oil obtained by hydro-distillation (HD), with the results shown in Table 1 and Table 3. The most represented class of molecules in the HD leaf EO is the oxygenated monoterpenes (53.5%). Geranial and neral exhibit a significant presence in HD leaf essential oils (EOs), while geranyl and neryl acetates account for 3.94% and 1.48%, respectively. Their respective alcohols, however, are present in quantities lesser than 1.5% in the HD leaf EOs. Sesquiterpene hydrocarbons constitute 3.04% of the HD leaf EOs, with spathulenol being the most abundant among them at 1.22% [25]. In Table 5, we compare the relative abundance (RA %) of the principal phytochemicals found in the HD leaf EO, as reported by Flamini et al. [25], with the relative abundance of molecules obtained in a steam-distilled essential oil from pompia leaves, characterized by Usach et al. [45], using liquid injection in a gas chromatographic device. According to Table 5, limonene, linalyl acetate, geranial, (E)-β-ocimene, linalool, and citral are the main components of the essential oil. Citral (the sum of geranial 11.1% and neral 6.8%) represents 17.9% of the essential oil and is primarily responsible for the antimicrobial properties of pompia essential oil [44].

Fancello et al. [40] collected additional pompia leaves in May–June near Siniscola, Sardinia, Italy. These leaves were suspended in water and subjected to steam distillation using a Clevenger-type apparatus. The qualitative GC-MS analysis of leaf EOs showed diverse results compared to previous literature. Specifically, the total ion chromatogram (TIC) from a full scan investigation revealed 27 molecules, including terpinen-4-ol, linalyl acetate, and some sesquiterpene derivatives [25,44] (Table 4). In the pompia leaf EO, the major components that differentiated these results from those presented in the literature (Flamini et al. [25]) were primarily trace compounds, except for linalyl acetate, which had a concentration of 300 mg/mL. Interestingly, Fancello et al. [44] revealed Δ2-carene in a significant quantity (nearly 22 mg/mL), whereas it was not found in their 2020 study. In the 2017 study, linalyl acetate (the acetic ester of linalool) was the main molecule (298.65 mg/mL), followed by limonene at about 257 mg/mL. Neral and geranial (citral isomers) were detected at lower concentrations compared to those indicated in the 2017 study by Fancello et al. [44], with concentrations of 87 and 98 mg/mL, respectively, compared to 173 and 214 mg/mL. A direct comparison of these data with those obtained by Flamini et al. [25] is not possible due to the use of different quantification methods. Table 4 summarizes the principal molecules identified in the pompia leaf EO in the studies by Fancello et al.

### 6.4. Leaf Volatiles

Fancello et al. [43] studied the essential oil of pompia leaves (PLEO) and its gaseous phase. PLEO fumigation was conducted by adding a measured amount of essential oil into a Petri dish using a device previously described by Petretto et al. [46]. The qualitative chemical analysis of the compounds released into the headspace was performed using HS-SPME/GC-MS (headspace solid-phase microextraction coupled with gas chromatography–mass spectrometry). Chemical analyses were conducted at two different time points, and the results indicated that the concentration of more volatile molecules was higher in the steam phase compared to the pure oil. These volatile components including myrcene (10.47 mg/mL), limonene (256.87 mg/mL), and other monoterpenes eluted in the initial part of the chromatogram (i.e., those with shorter retention times). However, less volatile components such as linalyl acetate (298.65 mg/mL), neral (86.81 mg/mL), geranial (98.39 mg/mL), and the sesquiterpene fraction were present in the steam phase. These findings were confirmed by qualitative analysis of the residue obtained by washing the Petri dishes with hexane after the HS-SPME/GC-MS analysis [43].

### 6.5. Peel Volatiles

Petretto et al. [4] examined the flavedo of eight different citrus species using HS-SPME-GC (headspace solid-phase microextraction coupled with gas chromatography) analysis. In the HS fraction of pompia flavedo, the main compound is limonene, which constitutes 94.1% of the total HS, similar to all other studied citrus species. The species most distinct from pompia in terms of volatile composition is *Citrus medica* [4].

Interestingly, Mignani et al. [3], found contrasting results using DNA markers such as AFLP (Amplified Fragment Length Polymorphisms), RAPD (Random Amplified Polymorphic DNA), and SCAR (Sequence Characterized Amplified Region) analysis. They found that pompia samples are genetically related to both *Citrus limon* and *Citrus medica*. However, the chemometric analysis of VOC (Volatile Organic Compound) composition in the HS fraction suggests that pompia samples are more closely linked to the group that includes *C. aurantium*, *C. sinensis*, *C. paradisi*, and *C. myrtifolia*.

### 6.6. Juice

Citrus fruits are extensively cultivated worldwide due to their high organoleptic and nutritional value [47]. They are popular and wholesome components of the human diet, offering numerous health benefits [48]. Citrus fruits provide an abundance of nutrients and phytonutrients. For instance, fresh and processed orange juice contains high levels of vitamins [3], minerals, and phytonutrients, including phenolic compounds [5,7] and carotenoids [8,9]. Citrus fruits are excellent sources of potassium, ascorbic acid, folate, polyphenols, and flavonoids (such as naringin, hesperidin, neohesperidin, citronin, narirutin, carotenoids, and polymethoxylated flavones) [5,33,34,38].

Potassium can lower blood pressure [36], while ascorbic acid (vitamin C) is vital for the immune system, protecting endothelial cells and low-density lipoproteins (LDLs) from oxidative stress [42] and generally acting as an antioxidant to reduce the risk of atherosclerosis [38]. Olic acid (vitamin B9) helps the body convert carbohydrates into glucose for energy and is essential for proper brain function, mental and emotional health, and the production of DNA and RNA. This is particularly important for rapidly growing tissues, such as during pregnancy, where it helps prevent neural tube birth defects, including cleft palate, spina bifida, and brain damage. Folic acid may also reduce the risk of heart disease by working with vitamins B6 and B12 to lower homocysteine levels [39], although supplements have not been found to be preventative [40].

Flavonoids, common in fruits and vegetables, have antioxidant and anti-inflammatory properties that protect LDL from oxidation, preventing atherosclerosis [24]. Carotenoids, such as beta-carotene, lycopene, lutein, and zeaxanthin, provide health benefits by reducing the risk of certain cancers and eye diseases due to their antioxidant properties and role as pro-vitamin A compounds [41]. Citrus fruits are also favored for their flavor. Beyond sugars and acids, the complex aroma of oranges arises from 36 aroma-active components, with no single character-impact compound. As fruits mature over the harvest season, sugars and many volatiles, such as terpenes, aldehydes, esters, and ketones, generally increase, enhancing flavor. Conversely, acids, including ascorbic acid, and bitter limonoids (limonin and nomilin) decrease, while non-bitter limonin and nomilinic acid glucosides increase. Phenolic compounds (flavonoid glycosides and polymethoxylated flavones) and hydroxycinnamic acids also tend to increase over the season [49,50,51].

Citrus fruits are consumed fresh or transformed into juices, jams, candies, and various ingredients for sweets and desserts. They are rich in vitamin C (23.6–87 mg per 100 g fruit) [52] and phenolic compounds, especially flavonoids [37]. Due to this, scientific interest in the antioxidant effects of citrus fruits has significantly increased. Studies have shown a correlation between citrus fruit consumption and a reduced risk of developing neurological and cardiovascular diseases, obesity, diabetes, and certain types of cancer [53,54].

Traditionally, pompia juice has been considered a waste product due to its organoleptic characteristics. There is only one study on pompia juice in the scientific literature. Barberis et al. [55] analyzed pompia juice and found that its yield is very low (12.3 ± 2%), the pH is 2.3 ± 0.5, the titratable acidity is 6.9 ± 0.2% citric acid equivalents, and the total soluble solids (TSS) are 7.2 ± 0.3 Brix. The flavor is notably sour and bitter.

After HPLC liquid chromatography, the authors identified a quinic acid derivative as the major chromatographic peak in pompia juice, although its quantification was not reported due to difficulties characterizing the compound more precisely. The phenolic composition of pompia juice is more similar to that of lemon juice than orange juice. Additionally, the primary flavonoid identified in pompia juice is chrysoeriol-6,8-di-C-glucoside (stellarin-2), first reported in a study on *Lemon cv Verna* juice [56]. This molecule has not been found in other juices.

Other compounds such as diosmin, isorhamnetin-7-rutinoside, and diosmetin-6,8-diglucoside are also prominent in pompia juice but significantly less common in lemon juice and absent in orange juice. The compound rhoifolin-4-glucoside is unique to pompia juice, whereas its aglycone, rhoifolin, is found in lemon juice. Hesperidin, responsible for the cloudiness in citrus juices [37], is present in all juices; it is found in very low concentrations in pompia juice but is most prevalent in various orange species. Notably, anthocyanins were not detected in pompia juice. 

The phenolic characterization of pompia juice provides valuable information but does not fully resolve the mystery surrounding pompia. Many compounds found in pompia juice are also present in lemon juice, such as flavonoids like hesperidin and diosmin [55]. Some compounds, like rhoifolin-4-glucoside, are typical of citron (*Citrus medica* L.) [49,57]. Others, such as eriocitrin and diosmin, are characteristic of bergamot [37,50], a species often described as a hybrid between sour orange and lemon [58]. Additionally, there are similarities between the essential oil compositions of pompia [44] and bergamot [59].

Table 6 summarizes the complete list of phytochemical constituents of pompia discovered in different plant parts, along with their chemical structures.

## 7. “Pompia Intrea” Candied Fruit Characterization

“Pompia Intrea” (PI), a traditional Sardinian culinary delicacy, is renowned for its richness in bioactive molecules with beneficial biological effects. Extracts obtained from PI, a traditional candied fruit made primarily from pompia, were described by Deiana et al. [6]. Analyzing the chemical characteristics of traditional foods for their health benefits is particularly relevant in the context of global market expansion [60,61,62].

In Deiana study, PI was prepared from pompia fruits gathered in the Baronia region of Sardinia in March 2017. Each batch of fresh pompia fruits was processed by various local specialists to obtain candied products following traditional procedures, with the addition of multifloral honey. Extracts were obtained according to the method described by Incani et al. [63] with minor modifications.

Using HS-SPME-GC (headspace solid-phase microextraction coupled with gas chromatography–mass Spectrometry) analysis, it was demonstrated that limonene was the predominant molecule in all PI samples, constituting 72.1–86.2% of the total volatile compounds. This aligns with previous findings that limonene is the principal compound in pompia’s flavedo, accounting for 94.1% of its volatile organic compounds, as revealed by headspace solid-phase microextraction [4]. Hence, the manufacturing conditions of PI did not significantly affect the richness of limonene, which originated from the fruit.

Limonene is a bioactive compound with a wide range of effects, including antioxidant [64], antimicrobial [65], and anticarcinogenic activity [66]. It also possesses antidepressant, spasmolytic, anxiolytic, gastroprotective, and immunostimulant properties [67].

The second class of molecules detected in the HS analysis were terpenes, which were significantly less abundant than limonene. This chemical profile resembles that of pompia’s flavedo, with monoterpenes such as β-myrcene (1.2–2.2%), trans-β-ocimene (0.3–1.8%), neryl acetate (0.5–1.9%), and geranyl acetate (0.4–1.4%), and sesquiterpenes such as trans-caryophyllene (0.3–1.5%) [4]. These same molecules—myrcene, cis-β-ocimene, neryl acetate, and geranyl acetate—were among the less abundant components of the pompia flavedo HS [4].

Cis-β-ocimene, geranial, neral, and myrcene exhibit various bioactivities, including analgesic, anti-inflammatory, antipsychotic, spasmolytic, hypnotic, sedative, and muscle relaxant properties [68,69]. The most represented sesquiterpene was trans-caryophyllene, which was also isolated in the headspace (HS) of pompia’s flavedo [4]. Other sesquiterpenes detected included β-bisabolene and trans-α-bergamotene, which were also found among the minor HS constituents of pompia’s flavedo, each with an abundance of less than 0.4% [4].

Different samples contained small percentages (less than 0.7%) of benzene derivatives, such as benzaldehyde and phenylacetaldehyde. These compounds were not present in pompia’s flavedo [4], but they might be correlated with the honey used, as benzene derivatives are commonly found in honey [70]. It is possible that many volatile organic molecules from honey were missed due to the conditions of the PI preparation. Aliphatic molecules such as hexadecanoic acid, heneicosane, and tricosane, likely originating from the honeycomb environment, were found in the headspace [70].

The only heat-derived molecule present in all samples was furfural. Compounds like 2-furanmethanol, 2,3-dihydro-3,5-dihydroxy-6-methyl-4H-pyran-4-one, and 5-hydroxymethylfurfural were found in some samples. However, the HS-SPME technique is less suitable for identifying volatile heat artifacts (e.g., 5-hydroxymethylfurfural) in honey-based products compared to the solvent extraction technique [70].

Additionally, flavonol glycosides were identified in the PI extract. Various apolar non-glycosidically linked polymethoxylated flavonoids (PMFs) with notable antineoplastic effects have been observed in Citrus species. Polar phenolic metabolites, such as di- and tri-methoxylated flavonols like limocitrol- and limocitrin-β-D-O-glucosides with HMG-substitutions (3-Hydroxy-3-MethylGlutaric acid), were also found. These HMG-substituted derivatives are noteworthy due to their unexpected statin-like properties [71].

The LC-DAD analysis (liquid chromatography with a diode array detector) was used to quantify the most typical phenolic compounds. Neohesperidin, neoeriocitrin, and naringin were the most concentrated molecules (45.7 ± 11.1, 27.8 ± 6.5, 21.5 ± 4.3 mg/L, respectively). These molecules were also predominant in the pompia rind extracts used to formulate innovative phospholipid vesicles aimed at preventing oxidative stress in skin cells [34].

Various glucosidic derivatives of dihydroferulic acid were found, with dihydroferuloyl–O-glucoside being the most represented (15.8 ± 7.2 mg/L, expressed as ferulic acid equivalent). The most abundant flavonoid was quercetin 3-rutinoside 7-glucoside (8.8 ± 2.7 mg/L, expressed as quercetin-3-O-rutinoside equivalent). A significant quantity of 5-(hydroxymethyl) furfural (HMF) was also detected (40.8 ± 23.5 mg/L). HMF is a typical product of sugar thermal degradation from both sucrose and honey sugars [72,73]. HMF, along with furfural and 2-furanmethanol, was also identified in the volatile fraction. The production of these furfural derivatives should be monitored due to their potential toxic effects on humans [74]. Therefore, strategies to limit their presence in PI are recommended, particularly during cooking processes when their production is promoted by high temperatures.

## 8. Potential Health Benefits: Antioxidant Activity of Pompia

The unstable atoms known as free radicals, particularly Reactive Oxygen Species (ROS), are commonly generated within the human body through various biochemical reactions. These free radicals can cause cellular damage through oxidation, which is linked to diseases such as cancer, atherosclerosis, Parkinson’s disease, and Alzheimer’s disease. Food products containing -OH (hydroxyl) functional groups are considered to have antioxidant potential, preventing oxidative damage by donating their electrons to free radicals [75]. Due to the perception that natural antioxidants are safer than synthetic drugs, they are increasingly recognized as better alternatives for designing new therapeutic approaches [76,77,78,79,80].

In this context, pompia has been the subject of several scientific studies due to its potential health benefits. Pompia essential oils derived from its leaves exhibit good antioxidant activity, consistent with previous results obtained on *Citrus* spp. [42]. Considering that limonene is the major compound of pompia essential oil, these results are confirmed by Bacanlı, et al. who described the antioxidant activity of this compound [81]. Moreover, it is reasonable to assume that neral, another molecule in pompia and one of the most potent scavenging compounds, also contributes to antioxidant activity [82]. However, in contrast with previous works, the study by Flamini et al. [25] reported that pompia showed weak antioxidant activity determined by the 2,2-diphenyl-1-picrylhydrazyl (DPPH) assay. Pompia peel essential oil samples presented IC50 values of 39.33 ± 6.1 mg/L, while ascorbic acid (used as a positive control standard) had IC50 values of 4.70 ± 0.86 mg/L (26.6 µM).

The antioxidant substances in “Pompia Intrea” (PI) extracts were evaluated, particularly the total phenol (TP) and total flavonoid (TF) contents [6]. The antioxidant activity of the PI extracts was measured by FRAP, CUPRAC, ABTS•+, and DPPH• assays, showing average values of 8.60 ± 5.29 mmol Fe^2+^/L, 14.25 ± 5.38 mmol Fe^2+^/L, 1.48 ± 0.37 mmol TEAC/L, and 4.62 ± 0.94 mmol TEAC/L, respectively. On average, the total polyphenol content and antioxidant capacity of PI were comparable to those of several other candied fruits, such as quince [83], plums, apricots, figs, and dates [84]. Some of the most concentrated phenols in PI, such as naringin and neohesperidin, have been studied for their protective effects due to their high antiradical scavenging activity [81,85]. Moreover, neoeriocitrin, found in PI in relevant concentrations, proved to be the most effective among the citrus polyphenols tested in preventing lipid peroxidation [86].

Barberis et al. [55] sought to understand the relationship between total phenolic content and phenolic contribution to antioxidant activity. The antioxidant activity content in pompia is higher than in lemons and oranges, making its contribution to antioxidant capacity significant. The authors measured the redox potential, which indicates the reducing power in pompia juice, containing different classes of compounds, highlighting the importance of the class of molecules and their relative quantities [87]. The antioxidant activity occurs at a very low potential, close to zero mV [88,89,90], which differs significantly from oranges and lemons. These differences should be attributed to the activity of phenolics with different redox potentials.

The ability of the juices to exert an antioxidant action was further tested in a more biologically relevant experimental system using cell cultures. Differentiated Caco-2 cells were chosen as a model of human enterocytes. Control experiments first assessed potential juice cytotoxicity, showing unchanged cell viability in the presence of all tested juices, compared to untreated cells (100% viability), in the concentration range of 10–500 µg/mL. The potential protective effect of the juices against TBH-induced oxidative damage in Caco-2 cell monolayers was then investigated. A significant increase in ROS production was caused by 2.5 mM TBH, compared to untreated cells (control), starting from 15 min of incubation. TBH generates ROSs that catalyze the peroxidation of membrane lipids [91]. In the presence of the juices, ROS production significantly decreased starting from 50 µg/mL compared to the positive control (TBH 2.5 mM alone) and gradually lowered as the juice concentrations increased. At 500 µg/mL, pompia and lemon juices brought ROS production back to the untreated cells’ level. Orange juices appeared much less effective. The ability of the citrus juices to protect Caco-2 cell monolayers against TBH oxidative injury was also assessed by measuring malonyldialdehyde (MDA) levels after 2 h of incubation with 2.5 mM TBH, when advanced peroxidation processes damaged the membrane lipid fraction, giving rise to detectable oxidation products like MDA [92]. A 2.5-fold increase in MDA levels compared with control cells was observed in the culture medium of TBH-treated cells. Treatment with the juices significantly inhibited MDA production starting from 10 µg/mL for Sanguinello and Lemon and from 50 µg/mL for all juices.

These data indicate that pompia juice is highly effective against ROS production and its damaging effects, consistent with the capacity of pompia rind extracts to prevent oxidative damage and promote the viability of human keratinocytes and mouse fibroblasts [34]. The antioxidant activity of pompia juice in cell cultures may be related to its radical scavenging ability. It is noteworthy that pompia juice, despite showing the best scavenging ability in chemical assays, did not protect cell membranes more than the other juices. These results further support the opinion that the action of an antioxidant greatly depends on the reaction environment and may be exerted through multiple mechanisms in a complex environment such as a biological system.

Overall, the analyzed studies highlight pompia’s potential as a source of natural antioxidants, with promising applications in healthcare and food preservation.

## 9. Conclusions

*Citrus limon var. pompia Camarda var. nova*, commonly known as pompia, is a unique citrus ecotype from Sardinia with notable botanical, phytochemical, and health-related properties. However, the morphological, phytochemical, and genetic characteristics of pompia are unique, along with its close affinities to the *Citrus limon* s.l. complex, particularly its polyembryony and generally fertile seeds.

This extensive review highlights the complex phytochemical profile of pompia, which includes essential oils rich in limonene (82.1%), geranial (1.7%), neral (1.4%), and various flavonoids. These compounds contribute to the fruit’s significant antioxidant, antimicrobial, and potential anti-carcinogenic activities. Studies have demonstrated the antimicrobial efficacy of pompia essential oils and juice against a range of bacterial strains, including *Staphylococcus aureus* and *Escherichia coli* [25,55], as well as its ability to inhibit biofilm formation, a property particularly relevant for treating resistant infections [55]. Additionally, the incorporation of pompia extracts into nanocarriers such as liposomes and phospholipid vesicles has shown promising results in enhancing the delivery and efficacy of these bioactive compounds, particularly for skin and mucosal infections [45,93,94]. The traditional uses of pompia in Sardinian cuisine, particularly its incorporation into desserts and beverages, underline its cultural and economic significance. The chemical analysis of pompia’s various parts—peel, leaves, rind, and juice—reveals a rich array of bioactive compounds, making it a valuable candidate for further research and development in nutraceuticals and natural health products.

## 10. Future Perspectives

The comprehensive phytochemical and biological profile of pompia opens several avenues for future research and application. Future studies should aim to optimize extraction methods to yield higher concentrations of bioactive compounds, with advanced techniques such as supercritical fluid extraction and high-pressure processing being explored to enhance efficiency and purity. Investigating the molecular mechanisms underlying the antioxidant and antimicrobial activities of pompia compounds could provide deeper insights into their health benefits and potential therapeutic applications. Conducting clinical trials to evaluate the efficacy and safety of pompia extracts and formulations in humans is crucial, as this could pave the way for the development of new natural health products and dietary supplements. Exploring sustainable cultivation practices and genetic improvement strategies to enhance the yield and quality of pompia fruits could support its commercial production and availability. Additionally, the potential of pompia extracts in food preservation, functional foods, and cosmetic formulations warrants further investigation, as their natural antioxidant and antimicrobial properties could offer alternatives to synthetic additives. Establishing standardized protocols for the extraction, characterization, and quality control of pompia products is essential for regulatory approval and market acceptance. By addressing these future perspectives, pompia could significantly contribute to the fields of natural medicine, food science, and biotechnology, enhancing both human health and economic development in regions where it is cultivated.

## Figures and Tables

**Table 4:** Main components found in the leaf EO of *Citrus limon* var. *pompia*. Comparison between the results of the works of Fancello et al. 2020 [43] and Fancello et al. 2017 [44], expressed as mg/mL.

Compound	Fancello et al., 2020—mg/mL [43]	Fancello et al., 2017—mg/mL [44]
Linalyl-acetate/geraniol	298.65	256.30
Limonene	256.87	-
Geranial	98.39	213.8
Neral	86.81	172.9
Myrcene	10.47	15.3
Nerol	5.47	37.5
Linalool	84.13	8.5
Geranyl acetate	-	18.9
Epten-2-one(6-methyl)	-	13
α-Phellandrene	7.07	-
γ-Terpinene	7.08	-
Z beta O-cymene	-	8.0
E beta O-cymene	52.77	71.7
Terpinolene	14.47	3.3
Citronellal	13.85	5.4

**Table 5:** Main compounds detected in the essential oil from pompia leaves. Comparison between results of works of Flamini et al. [25] and Usach et al. [45] expressed as relative percent area. RA, Relative Abundance (%).

Compound	Flamini et al., 2019 RA% [25]	Usach et al., 2020 RA% [45]
Limonene	28.64	29.7
Trans-β-ocimene	10.50	4.4
Linalool	0.56	11.0
Citronellal	1.27	0.2
Nerol	1.49	2.9
Neral	18.84	6.8
Geranial	24.44	11.1
Geranyl acetate	3.94	-
Linalyl acetate	-	20.9
α-Terpineol	41.18	8.4
Neryl acetate	13.56	5.5
Cariophyllene	-	1.3
Carene	-	21.7
Chrysanthenol cis	-	3.0
Verbanol iso	-	10.8

**Table 6:** Complete list of phytochemical constituents of *Citrus limon var. pompia Camarda var. nova* in different plant parts and their chemical structures.

Compound	Chemical Class	Chemical Structure	Plant Part
Rhoifolin 4-glucoside or apigenin 7-O-neohesperidoside 4-glucoside	Flavonoids	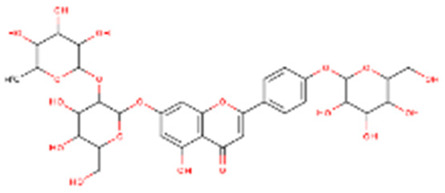	juice
Delta-2- carene	Terpenoids	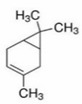	E.O. leaf
Stellarin-2 or chrysoeriol 6,8-C-glucoside	Flavonoids	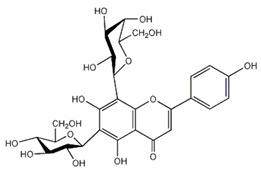	juice
Diosmetin 6,8-diglucoside	Flavonoids	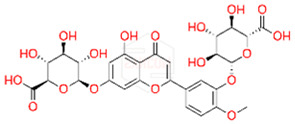	juice
Diosmin	Flavonoids	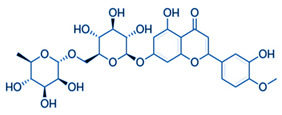	juice
Eriocitrin	Flavonoids	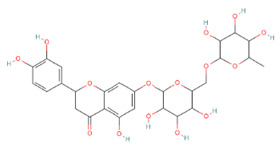	rind extract
Ferulic acid	Terpenoids	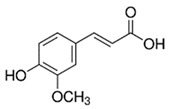	rind extract
Gallic acid	Terpenoids	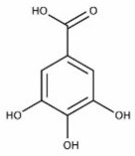	rind extract
Geranial	Terpenoids	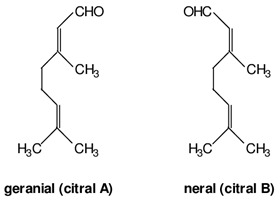	E.O. leaf and E.O. rind
Neral	Terpenoids		E.O. leaf and E.O. rind
Geranyl acetate	Terpenoids	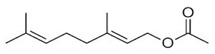	E.O. leaf
Isorhamnetin 3-o-rutinoside	Flavonoids	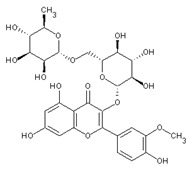	juice
Limonene	Terpenoids	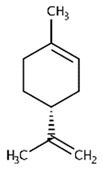	E.O. leaf and E.O. rind
Linalil-acetato/geraniolo	Terpenoids	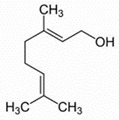	E.O. leaf
Linalool	Terpenoids	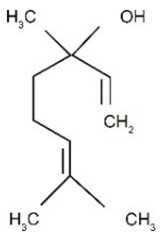	E.O. leaf and E.O. rind
Myrcene	Terpenoids	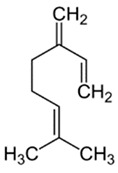	E.O. leaf and E.O. rind
Naringin	Flavonoids	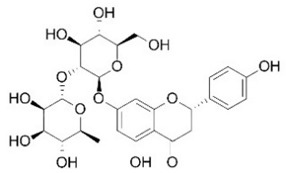	rind extract
Neoeriocitrin	Flavonoids	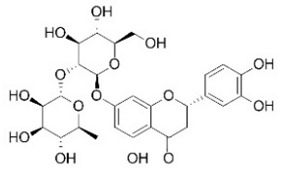	rind extract
Nerol	Terpenoids	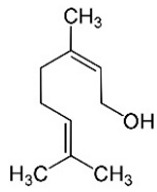	E.O. leaf
(e)-βeta-ocimene	Terpenoids	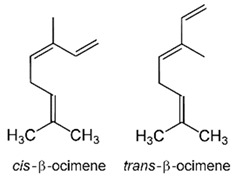	E.O. leaf and E.O. rind
(z)-βeta-ocimene	Terpenoids		E.O. leaf and E.O. rind
Hesperidin	Flavonoids	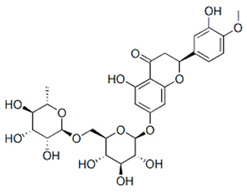	rind extract and juice
Neohesperidin	Flavonoids	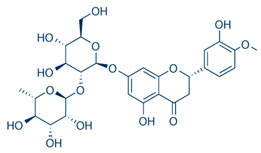	rind extract
Myricetin-3-galactosyde	Flavonoids	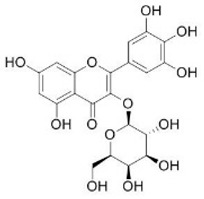	rind extract
Quinic acid	Terpenoids	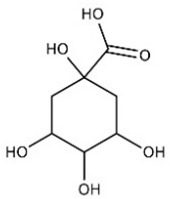	rind extract
Robinin	Flavonoids	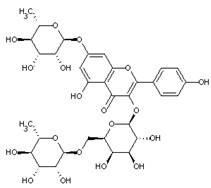	rind extract
Rutin	Flavonoids	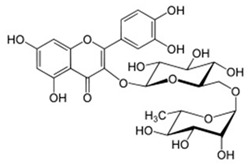	rind extract
Sinapic acid	Terpenoids	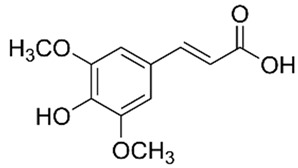	rind extract
α-Terpineolo	Terpenoids	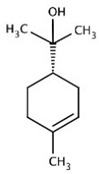	E.O. leaf

## Data Availability

All relevant data are available within the manuscript.

## List of Abbreviations

CAT	Catalase
CP	Cold pressing
EO	Essential oils
GC-FID	Gas Chromatography-Flame Ionization Detection
GC-MS	Gas chromatography–mass spectrometry
HS-SPME-GC	Headspace solid-phase microextraction coupled to gas chromatography
HD	Hydro-distillation
HMF	Hydroxymethyl-furfural
HMG	3-Hydroxy-3-MethylGlutaric acid
HPLC–MS	High-performance liquid chromatography–mass spectrometry
HS	Headspace
PI	Pompia Intrea
PLEO	Pompia leaves essential oil
PMF	Methoxylated flavonols
POD	Peroxidase
PPO	Polyphenol oxidase
SOD	Superoxide dismutase
TIC	Total Ion Chromatogram
TSS	Total Soluble Solids
VOCs	Volatile Organic Compounds
